# 3D quantification of metal-induced geometric distortions in MRI

**DOI:** 10.1038/s41598-025-90645-5

**Published:** 2025-02-28

**Authors:** Hao Li, Ali C. Özen, Alexander Juerchott, Michael Breckwoldt, Jessica Jesser, Dominik F. Vollherbst, Daniel Schwarz, Martin Bendszus, Sabine Heiland, Tim Hilgenfeld

**Affiliations:** 1 Department of Neuroradiology, University Hospital Heidelberg, Im Neuenheimer Feld 400, 69120 Heidelberg, Germany; 2 Department of Radiology, Medical Physics, Medical Center, Faculty of Medicine, University of Freiburg, Freiburg, Germany

**Keywords:** Magnetic resonance imaging, Susceptibility artifacts, Metallic implants, Geometric distortion, Image noise, Accuracy and reliability, Magnetic resonance imaging, Three-dimensional imaging

## Abstract

The increasing number of patients with metal implants raises concerns about metal-induced geometric distortions (MD) in MR-guided treatments. This study proposes a method for three-dimensional quantification of MD and evaluates its accuracy and reliability. A 3D lattice phantom was designed and measured with two sequences (VIBE and SPACE) and two implants (crown-supported-dental-implant and stainless-steel-bracket). Automated detection of displacement of 9360 crossing points caused by MD was performed. Distortion-quantification accuracy was improved by correcting for noise-induced error (NE), related to different signal-to-noise ratios (SNR), and implant-related signal loss and pile-up artifact volumes (SLPUA). The method’s accuracy was validated against computed tomography. Results showed high reliability, with an excellent intraclass correlation coefficient (≥ 0.99) and low mean residual errors in all directions (2.6%/1.6%/1.8% of voxel size in X/Y/Z direction). SNR/SLPUA volumes were significant confounders (p-value ≤ 0.001) when comparing different sequences/implants, but corrections significantly reduced their impacts (p-value ≤ 0.001). This method enables accurate 3D MD quantification and fair comparison across different sequences/implants. By optimizing MRI protocols for MD minimization and defining implant-specific MD profiles for patient data correction, it may help improve spatial accuracy in MRI-guided treatments in the future.

## Introduction

Magnetic resonance imaging (MRI) plays an essential role in supporting clinical diagnosis and treatment due to its high soft tissue contrast and non-ionizing character. However, susceptibility artifacts caused by metallic implants can severely downgrade image quality. Susceptibility artifacts consist of signal loss and pile-up artifacts (SLPUA), as well as geometric distortion (GD)^1^. Current research mainly focuses on the evaluation of SLPUA generated by various metallic implants^2–6^. However, metal-induced GD (MD) is also an essential factor since it affects the spatial accuracy of MR images, and thereby the spatial accuracy of target regions, -volumes and organs at risk. That is of paramount importance for MRI-guided treatment, such as radiotherapy^7–12^ and navigated surgery in various disciplines^13^. For instance, MRI-based planning for intracavitary brachytherapy in cervical cancer allows for improved tumor delineation, leading to better local tumor control and increased overall survival^14,15^. Consequently, more research is necessary to improve the spatial accuracy of MRI in patients with implants in terms of distortions. This can be addressed firstly by developing a method that allows for optimizing MRI sequences and sequence parameters for minimal distortions and secondly by defining implant-specific distortion profiles experimentally that can then be applied to patient data to further minimize implant-related distortions. These advancements may improve treatment planning accuracy by ensuring precise tumor targeting and organ delineation, reducing safety margins to protect healthy tissues, and facilitating the reliable integration of MRI into ART workflows, optimizing patient outcomes and minimizing treatment risks^8,11,16,17^. Published studies on GD predominantly evaluated the MRI system dependent effects e.g., caused by static-field inhomogeneities^12^ or the nonlinear gradient fields^18,19^. However, the results are insufficient for the in-vivo situation as factors originating from the patient must be considered as well. Some available studies on patient dependent factors focus on GD due to anatomic interfaces^8,20,21^. Studies evaluating the additive negative effect of the metallic implants on MD, which can be often clinically encountered^4,12,22,23^, are even more rare^24^. The latter may affect the outcome of radiotherapy by decreasing accuracy of CT-MR registration, highlighting the clinical importance of these artifacts^12,25^. Up to now, it is unclear whether advantages of metal-artifact reduction sequences for reduction of SLPUA^5,25–27^ also apply for MD reduction. To compare the efficacy of different sequences for MD reduction, an accurate and reliable quantification method is necessary. So far, MD in the whole field of view (FOV) is only quantified in one study, which quantified the MD induced by orthopedic devices^24^. However, the used methodology is limited for three reasons: First, only four layers are analyzed due to the phantom design and the employed sequences are optimized for musculoskeletal imaging with large slice thicknesses, resulting in an incomplete quantification of MD for the whole imaging space. Second, the effect of image acquisition parameters on the measurement accuracy of the method is unknown. The latter is of importance, as a previous study points out an effect of the signal-to-noise ratio (SNR) on the accuracy of the measured GD due to B0 inhomogeneity and gradient-fields non-linearity^28^. However, the influence of SNR difference is not analyzed and corrected. Consequently, any modification of sequence parameters or a comparison of different sequences will alter the accuracy of GD quantification, which may substantially alter results and thereby may adversely affect comparability. Third, SLPUA volumes of different sequences can differ substantially. Consequently, the areas and volumes that can be assessed for distortions will differ as well and limit comparability of the results and thereby sequences. To address these limitations, our study aims to develop an accurate and reliable method to spatially quantify the metal-induced GD in all directions for varying SNR and varying SLPUA volume. Moreover, it should be applicable to any types of sequences, metallic materials and body regions.

## Materials and methods

### Phantom design and sample materials

**Fig. 1:**
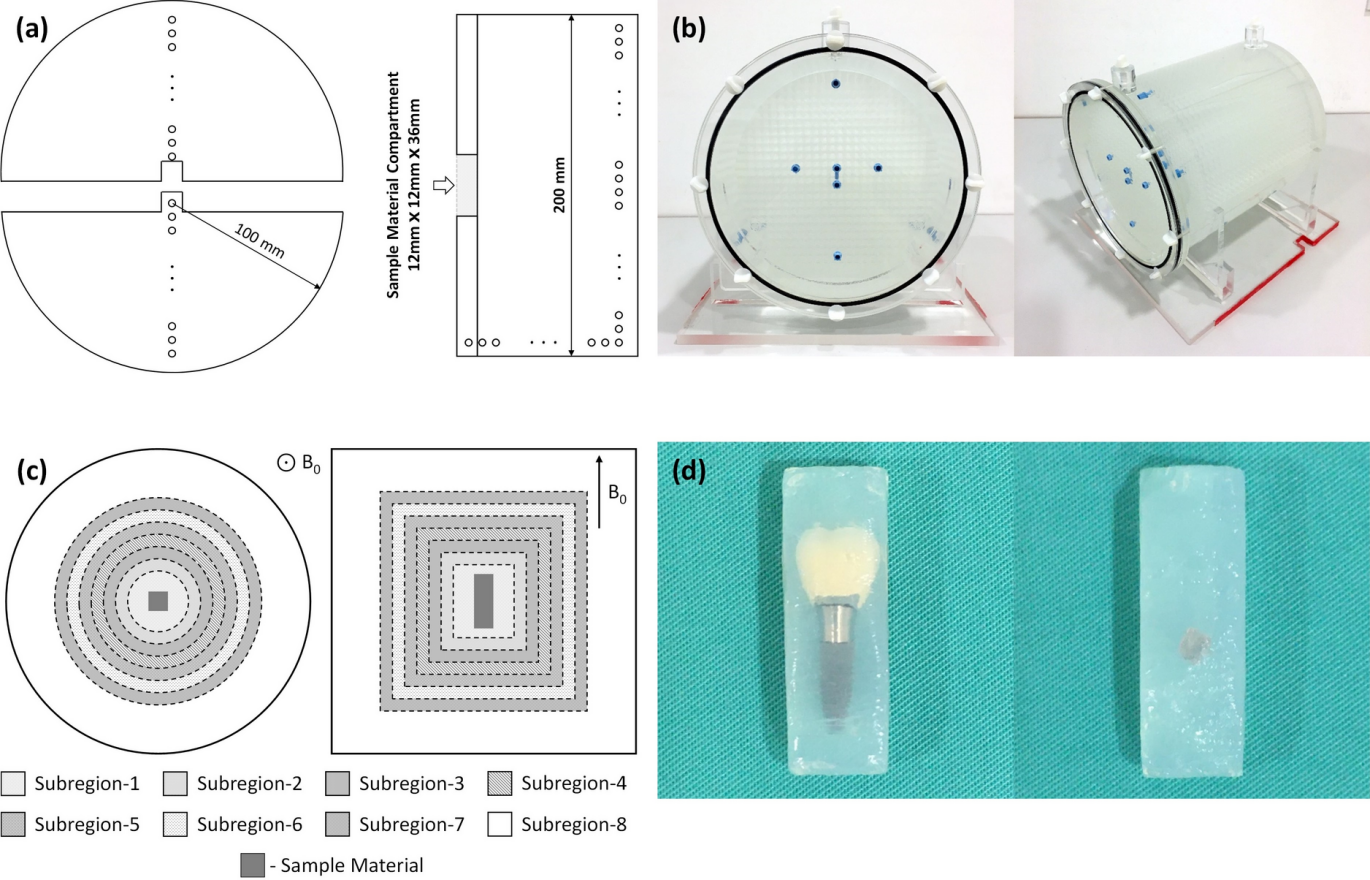
Illustration of phantom structure and metallic devices: (**a**) axial view of the phantom structure with the cylindrical phantom split into two segments and sagittal view of the bottom segment with marked compartment for sample materials; (**b**) photos of the 3D phantom; (**c**) axial and coronal views of the phantom showing subregions and compartment for sample material; (**d**) sample materials of non-precious alloy crown-supported titanium implant / stainless-steel bracket embedded in agarose.

A 3D lattice phantom was CAD/CAM-designed and built out of acrylic resin (115 mm in radius and 250 mm in length. Precision Mechanics Department of Heidelberg University, Heidelberg, Germany). The phantom consists of a cylindrical inner part (100 mm in radius and 200 mm in length) with capillary tubes in three orthogonal planes (Fig. 1a,b). The inner part has two segments: the bottom piece was fixed in the barrel, and the top piece was removable, allowing the metallic test devices to be placed in or removed from the phantom. For our measurements, the phantom was filled with pure water at room temperature. The capillary tubes have a diameter of 4 mm and a spacing of 4 mm between them in each dimension. The phantom provides 9360 crossing points that can be used for GD analysis.

A titanium dental implant (4.3 mm × 4.3 mm × 10 mm, Titanium based on ASTM F67, Nobel Replace, Nobel Biocare, Switzerland) was used, along with a supported single non-precious alloy crown (porcelain fused to non-precious alloy. Dentaurum, Germany) shown in Fig. 1d, left and a stainless-steel bracket (2.2 mm × 3.2 mm × 4.3 mm, AlSl Type: 303 Se. Ormco Europe, Netherlands) shown in Fig. 1d, right. These sample materials were chosen due to their frequent use and the artifacts they produce in head and neck imaging; furthermore, both materials exhibit different magnetic susceptibilities resulting in substantially varying artifact volumes (difference more than a factor of 70 based on artifact-affected crossing points). The sample materials were embedded in agarose gel (1% concentration, Select Agar™, ThermoFisher Scientific; Waltham, MA, USA.) The size of the embedded block was adjusted to the sample material compartment in the phantom and placed in the scanner parallel to the B0 direction to simulate the in-vivo situation. When sample material was not placed in the phantom, the sample material compartment was filled with water.

### Imaging protocols and measurements

The MRI images were acquired on a 3-Tesla MRI system (MAGNETOM Prisma, Siemens Healthcare GmbH, Germany) with a pair of surface coils (Small Flex Coil, Siemens Healthcare GmbH, Germany). To evaluate the sensitivity of the proposed method for differences in signal-to-noise ratios (SNR), five protocols of a volumetric interpolated breath-hold examination sequences (VIBE) with/without parallel imaging and different numbers of averaging were used. A second type of sequence (sampling perfection with application optimized contrasts using different flip angle evolution (SPACE)) with a comparable SNR to the VIBE sequence number 5 was employed to test whether the proposed method can be used for sequence comparisons with different signal loss and pile-up artifact volumes. The imaging parameters are shown in Table 1, with SNR measured following the NEMA standard^29^. A 1 mm isotropic resolution was chosen for its widespread clinical use and reasonable acquisition time. Automatic shimming and 2D geometric correction were applied to all measurements. Each analysis round was repeated for three times.

**Table 1:** Imaging protocols and SNRs.

	VIBE1	VIBE2	VIBE3	VIBE4	VIBE5	SPACE
TR/TE	$6.73ms/2.41ms$					$2000ms/21ms$
Number of averages	$1$	$1$	$1$	4	9	$1$
Resolution	$192\times 192\times 192$					
Voxel size	$1.0mm isotropic$					
Slice oversampling	$100\%$					
Phase oversampling	$10\text{\%}$					
Readout bandwidth	$520Hz/Voxel$					$521Hz/Voxel$
Parallel imaging factor	$3\times$	$2\times$	$\text{n}/\text{a}$	$\text{n}/\text{a}$	$\text{n}/\text{a}$	$\text{n}/\text{a}$
Flip angle / FA variation	$10$					$PD Var$
Turbo factor	$\text{n}/\text{a}$					$35$
SNR	$14.49\pm 1.05$	$18.64\pm 1.75$	$19.83\pm 1.95$	$32.48\pm 2.92$	$39.20\pm 4.48$	$36.25\pm 7.61$
Increment from VIBE1	$0$	$4.15$	$5.34$	$17.99$	$24.71$	$21.76$

### Automated calculation of geometric distortion

**Fig. 2:**
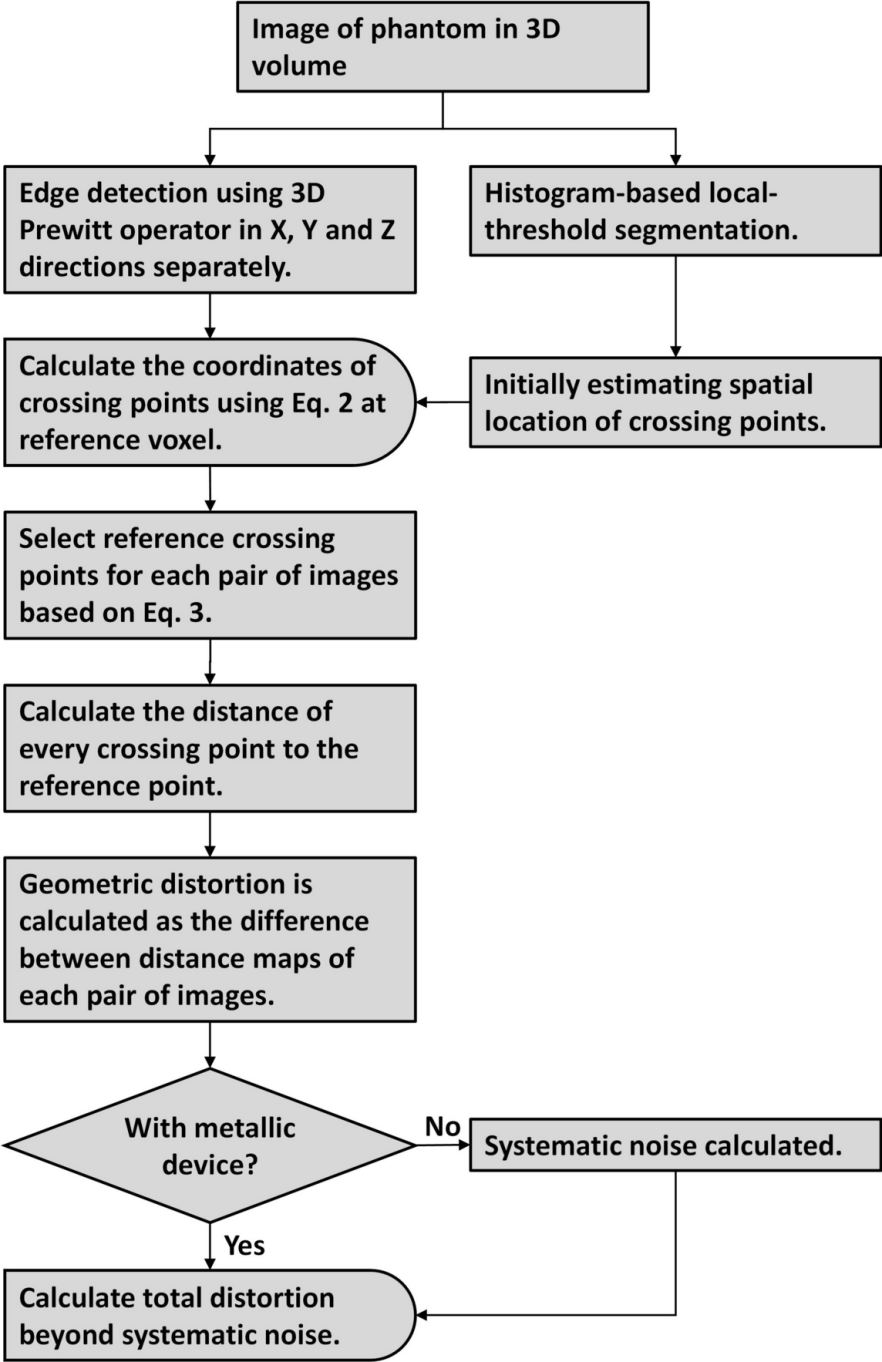
Flow chart of the automated calculation of the MD in the presence of a metallic device.

Figure 2 depicts the flow chart of the MD calculation. The MD were calculated using the spatial difference (Euclidean distance) of crossing points between measurements with and without a metallic device inside the phantom. The acquired images were processed using a custom automated algorithm programmed in MATLAB (2021a, MathWorks, Natick, MA).

To calculate the spatial location of each crossing point, an initial estimation was employed to obtain the crossing point voxels, where the calculation of the accurate spatial location of the crossing point was performed. The indices of crossing point voxels are computed after the segmentation of the grids in the images. Due to the non-uniformity of signal distribution caused by the sensitivity of coils in both the excitation and the receiving process, the global histogram-based segmentation would result in significant errors in the segmentation results. Thus, a histogram-based local-threshold segmentation was used in our experiments: an 8 × 8 window moved in the image from the top-left corner to the bottom-right corner with a stride of 8 and no padding. At each step, the image was segmented using the local threshold within the window, which was 0.95 times the mean signal intensity in the window.

The local threshold was computed as Eq. 1:

1 $$\begin{array}{*{20}c} {T = \alpha \frac{{\mathop \sum \nolimits_{i = 1}^{N} I_{i} }}{N}} \\ \end{array}$$

where $N$ is the number of voxels within the window and ${I}_{i}$ is the intensity of each voxel. $\alpha$ is the ratio factor assigned as 0.95 in this study to guarantee that the boundary voxels of the capillary tubes were correctly segmented. This value was chosen based on the severity of signal inhomogeneity. For binary segmentation, when signal distribution is more inhomogeneous, especially in grids with reduced signal intensity in some regions, a lower $\alpha$ is needed to prevent misidentification of grid voxels. Here, the background signal intensity was near zero, while the grid intensity was significantly higher. As a result, $\alpha$ was not highly sensitive to variations, and 0.95 consistently delivered optimal segmentation across all sequences.

In the segmentation label map, the central planes of tubes in each direction were calculated, and the crossing point voxels were the intersection points of three orthogonal planes. After the crossing point voxels were obtained, the spatial locations of the crossing points were calculated following the flow chart shown in Fig. 2. The sub-voxel spatial locations of the crossing points were measured using the first moments of the first derivatives of the signal intensity, and the first derivatives were calculated as the gradient maps of the images using 3D edge detection with 3D Prewitt operators in three directions^30^. The first moments were then calculated based on the gradient maps at the crossing point voxels and their neighboring voxels^30^:

2 $$\begin{array}{*{20}c} {M\left( {j,k} \right) = \frac{{\mathop \sum \nolimits_{p = 1}^{n} i_{p} \left| {g\left( {i_{p} ,j,k} \right)} \right|}}{{\mathop \sum \nolimits_{p = 1}^{n} \left| {g\left( {i_{p} ,j,k} \right)} \right|}}} \\ \end{array}$$

As an example of calculating the first moments in X direction, $M\left(j,k\right)$ is the calculated first moment in X direction, and $j$ and $k$ are the coordinates of the voxel in Y–Z plane. ${i}_{p} (p=1\ to\ n)$ represents the through-plane coordinate in X direction, and $g(i,j,k)$ represents the values in the gradient map in X direction. The first moments in Y and Z directions are calculated in the same manner.

To avoid the interference from the noise in the images, the first moments were computed at voxels within a 3 × 3 square window centered on the crossing point voxel, and the spatial location of a crossing point was computed as the average value of the first moments at all voxels in this window. After the above steps, the accurate spatial locations of all crossing points for each measurement had been obtained.

Before calculating the distortion between different measurements, an alignment of these measurements was needed since the original images of these measurements were not co-registered. A reference crossing point from each measurement was the starting point of the coordinate system of each measurement, then the coordinates of other crossing points could be calculated using the distance of the crossing points to the reference crossing point in three directions. With the same reference crossing point was selected among measurements, these measurements were aligned and the coordinate differences of all the other crossing points between different measurements were calculated. The criteria for selecting the reference crossing point for each repetition were that the distance maps’ minimal difference was obtained:

3 $$\begin{array}{*{20}c} {\arg \mathop {\min }\limits_{i} \mathop \sum \limits_{j} \left| {\left( {M_{j}^{\left( 1 \right)} - M_{i}^{\left( 1 \right)} } \right) - \left( {M_{j}^{\left( 3 \right)} - M_{i}^{\left( 3 \right)} } \right)} \right| + \left| {\left( {M_{j}^{\left( 2 \right)} - M_{i}^{\left( 2 \right)} } \right) - \left( {M_{j}^{\left( 3 \right)} - M_{i}^{\left( 3 \right)} } \right)} \right|} \\ \end{array}$$

where ${M}_{i}^{(1)}, {M}_{j}^{(1)}, {M}_{i}^{(2)}$ and ${M}_{j}^{(2)}$ are the first moments of *ith* and *jth* crossing point from the measurement with a metallic device and one of the measurements without a metallic device in X, Y, or Z direction, ${M}_{i}^{(3)}$ and ${M}_{j}^{(3)}$ are the first moments of *ith* and *jth* crossing point from the other measurements with a metallic device. Then the final coordinates of crossing points were computed as the distance between each crossing point and the reference crossing point.

The geometric displacement was characterized by the difference in the coordinates of crossing points:

4 $$\begin{array}{*{20}c} {du_{i} = u_{i}^{\left( 1 \right)} - u_{i}^{\left( 2 \right)} } \\ \end{array}$$

$$\left( {u = x,y,z\quad and\quad i = 1, 2, \cdots N} \right)$$

### Distortion evaluation in the presence of signal noise

Three repetitions, each of which consisted of one measurement with a metallic device and two without a metallic device acquired in a row, were performed for each sequence and each metallic device. The noise-induced error (NE) was the error caused by the image noise during the image-based calculation of distortion. The range of NE for each crossing point was calculated with pairs of measurements without a metallic device. The range of NE for each crossing point was defined as

5 $$\begin{array}{*{20}c} {du_{i}^{mean} = \frac{1}{M}\mathop \sum \limits_{j = 1}^{M} du_{i}^{\left( j \right)} } \\ \end{array}$$

6 $$\begin{array}{*{20}c} {du_{i}^{std} = \frac{{\sqrt {\mathop \sum \nolimits_{j = 1}^{M} \left( {du_{i}^{mean} - du_{i}^{\left( j \right)} } \right)^{2} } }}{M - 1}} \\ \end{array}$$

7 $$\begin{array}{*{20}c} {du_{i}^{{\left( {upper} \right)}} = du_{i}^{mean} + 3*du_{i}^{std} } \\ \end{array}$$

8 $$\begin{array}{*{20}c} {du_{i}^{{\left( {lower} \right)}} = du_{i}^{mean} - 3*du_{i}^{std} } \\ \end{array}$$

$$\left( {u = x,y,z\quad and\quad i = 1, 2, \cdots N} \right)$$

where ${du}_{i}^{\left(j\right)}$ is the geometric error of the *ith* crossing point calculated following Eq. 4 between a pair of measurements without metallic device from the *jth* repetition. ${du}_{i}^{(upper)}$ and ${du}_{i}^{(lower)}$ are the upper and lower limits of the NE of the *ith* crossing point, and $M$ is the number of repetitions of the measurements.

The metal-induced distortions (MD) were calculated between a measurement with a metallic device and its subsequent measurement without a metallic device. To calculate the MD, the crossing points with distortions in all directions beyond the ranges of the NE of each crossing point were included

9 $$\begin{array}{*{20}c} {Du_{i} = \left| {\max \left( {du_{i} - du_{i}^{{\left( {upper} \right)}} ,0} \right) + min\left( {du_{i} - du_{i}^{{\left( {lower} \right)}} ,0} \right)} \right|} \\ \end{array}$$

$$\left( {u = x,y,z\quad and\quad i = 1, 2, \cdots N} \right)$$

The MD can be, furthermore, divided into components in frequency encoding (MD-F), phase encoding (MD-P), and slice direction (MD-S). Therefore, the MD and MD-F / -P / -S were calculated as the sum of all crossing points’ MD:

10 $$Du = \mathop \sum \limits_{i = 1}^{N} Du_{i}$$

$$\left(u=x,y,z\right)$$

11 $$\begin{array}{*{20}c} {Dr = \mathop \sum \limits_{i = 1}^{N} \sqrt {Dx_{i}^{2} + Dy_{i}^{2} + Dz_{i}^{2} } } \\ \end{array}$$

### Distortion quantification in the volume of signal loss and pile-up artifacts

**Fig. 3:**
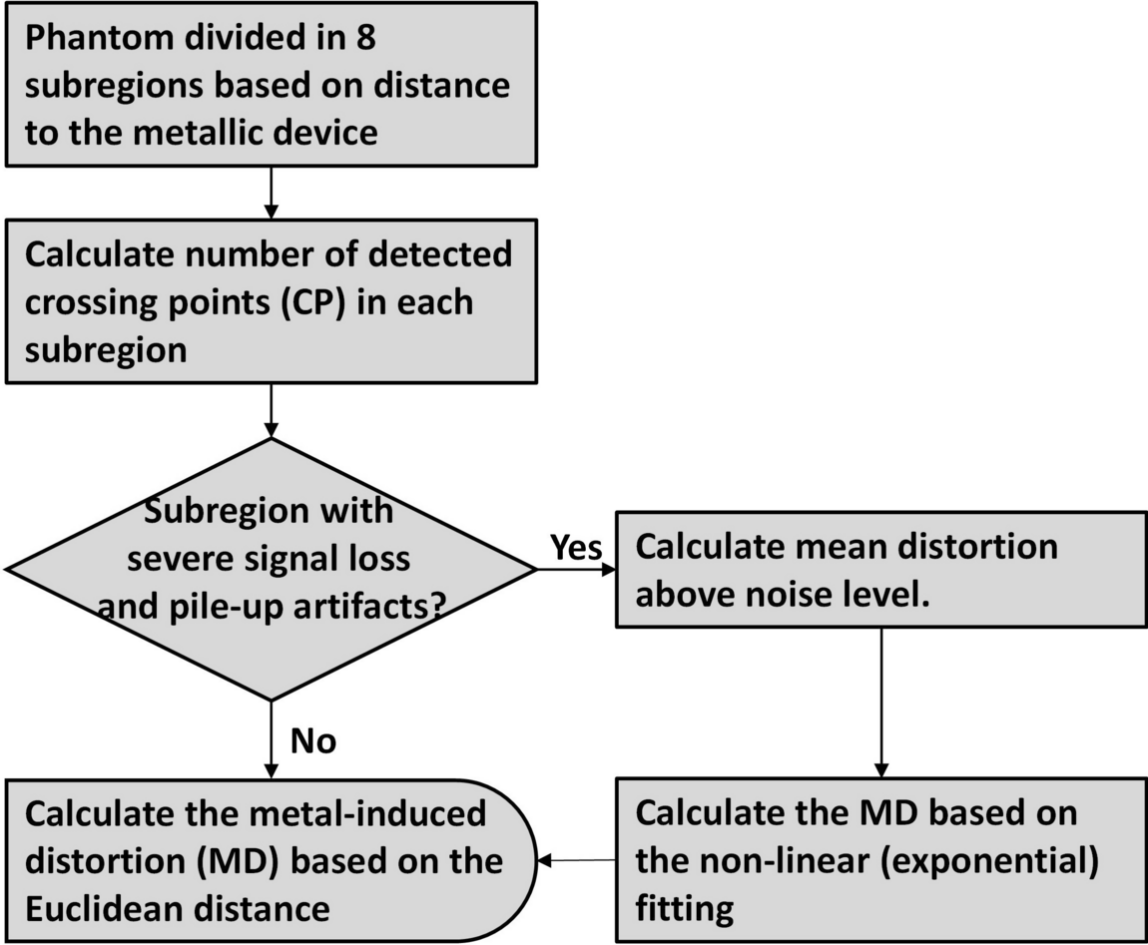
Scheme of mean MD estimation in the artifact-affected region.

The distortion in the volume of signal loss and pile-up artifacts was not measurable. However, as the volume of SLPUA varies significantly between sequences, a comparison of distortions outside these artifacts would have resulted in difference in the examined volumes and different minimal distances to metallic devices between sequences and, consequently, an unfair comparison. Therefore, it is necessary to estimate the distortion in the artifact-affected region as well, which did not allow for direct measurement of distortion. The distortion in the volume of SLPUA was quantified following the workflow described in Fig. 3.

For MD analysis, the phantom was divided into eight subregions centered around the sample material, with the same increment of 16 mm in diameter and 16 mm in length of the outer surface (except subregion-8, which was far away from the sample material with consequently minimized MD) (Fig. 1c). The average MDs of the subregions that were not severely affected by signal loss and pile-up artifacts (SLPUA) were calculated. Using the residual visible crossing points in the subregions with severe SLPUA for the analysis would result in underestimation of MD in these subregions. Therefore, it was necessary to estimate the mean distortion in subregions with most crossing points undetected. An exponential growth fitting was applied to predict the average MD in the subregions with the most severe SLPUA.

### Accuracy analysis

CT images of the phantom were acquired to evaluate the accuracy of the proposed algorithm. Three datasets of CT images (${I}_{CT}$) were acquired on a 64-slice CT scanner (Siemens Somatom, Siemens Healthcare GmbH, Germany) using a protocol with the resolution of $0.4\text{ mm}\times 0.4\text{ mm}\times 0.6\text{mm}$ at 335 mAs, 100 kV. These CT images were assumed as the ground truth, which were not affected by the metal induced distortion or the noise due to their extensive SNR. The density of the background (the substance of acrylic resin) and the foreground (the capillary tubes) were rescaled to ${I}_{rescale}$ to have the same mean intensities of the MRI images. Then, each rescaled CT dataset was added with Gaussian noise to generate a pair of datasets (${I}_{noise}$) with the same SNR as VIBE5. Following the proposed algorithm, the NE was calculated using pairs of ${I}_{noise}$ and removed from the GD calculated between ${I}_{rescale}$ and the ${I}_{noise}$ with NE correction process. The accuracy of the algorithm was finally evaluated using the residual NE between ${I}_{rescale}$ and the ${I}_{noise}$ after NE correction.

### Statistics analysis

Statistical analysis was done using GraphPad Prism (GraphPad Software, San Diego, CA) and MATLAB (2021a, MathWorks, Natick, MA). The two-way mixed effect intraclass correlation coefficient (ICC) was calculated to analyze the reliability of the measurement results and interpreted as described elsewhere^31^. Normal distribution of data was confirmed using the Shapiro–Wilk test. T-test, one-way analysis of variance (ANOVA) test and post hoc Tukey test were applied to test for the influence of SNR and distortion estimation. A descriptive p-value of ≤ 0.05 was considered statistically significant.

## Results

**Fig. 4:**
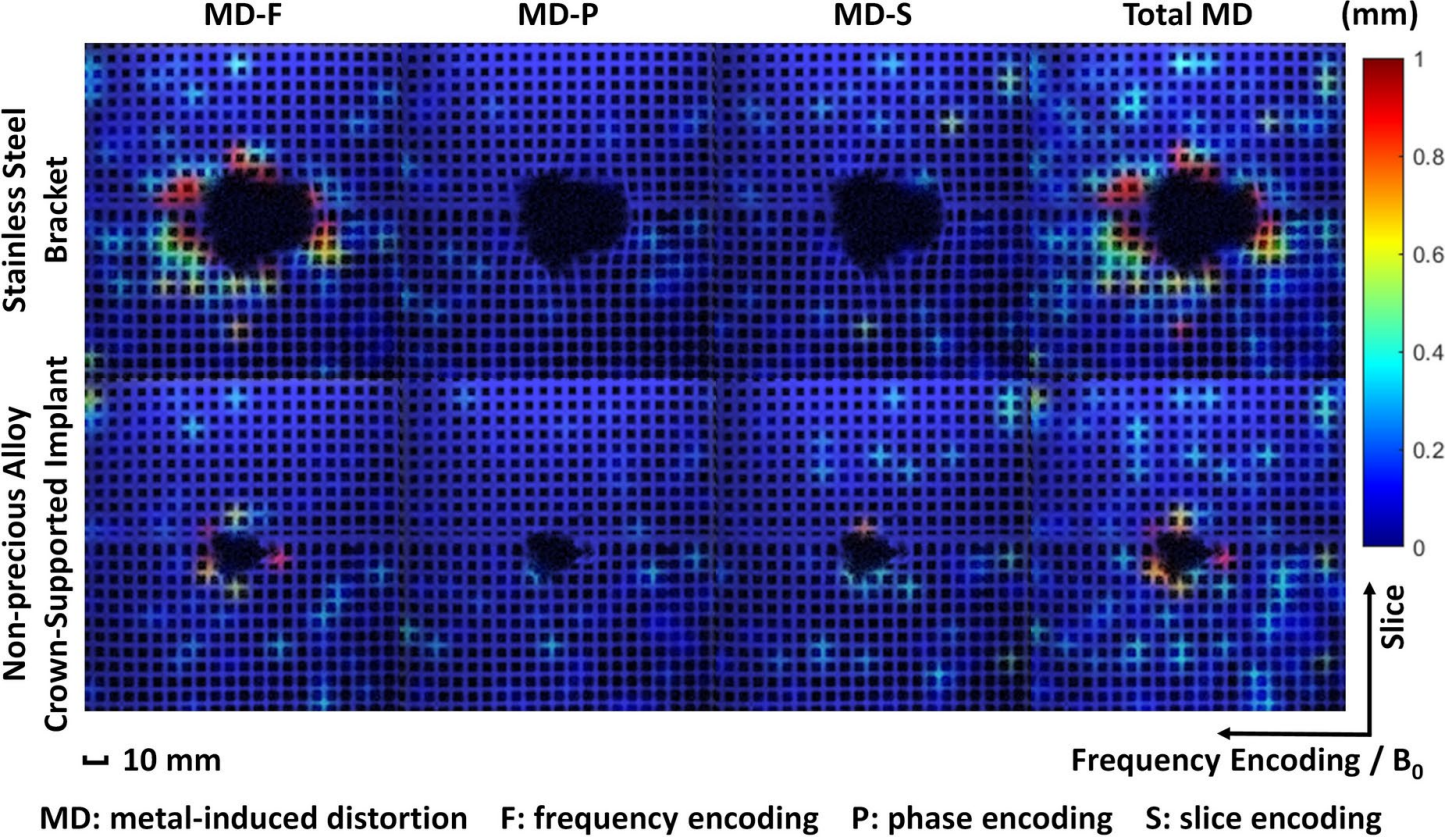
Sagittal view of VIBE 5 image with geometric distortion in color map: the sagittal images of VIBE 5 show bended grids near the signal-loss and pile-up artifact regions. The color map represents the severity of the calculated metal-induced distortion (MD) with the scale bar on the right. Left to Right: representative sagittal VIBE5 images with calculated metal-induced distortion in frequency-encoding/phase-encoding/slice direction (MD-F/P/S) and the total MD in Euclidean distances [mm].

The two types of metal samples produced different amounts of the MD in the phantom. As shown in Fig. 4, the sagittal images of VIBE 5 shows bended grids near the signal-loss and pile-up artifact region. The color map represents the severity of the calculated metal-induced distortion with the scale bar on the right. With the stainless-steel bracket, the MD was above 1 mm for the VIBE5 sequence in the region of 4.0 to 4.8 cm from the center of the implant, or 1.6 to 2.4 cm from the boundary of the signal loss region. With the non-precious alloy crown-supported implant only 1.6 to 2.4 cm and 0.8 cm, respectively, were observed. With both sample materials the frequency direction (MD-F) was the predominantly affected direction (Fig. 4).

### Reliability and accuracy analysis

Reliability assessment of the calculated MD with or without NE correction with both metallic materials resulted in ICC values above 0.99. Comparing MRI with CT as gold standard, the accuracy analysis resulted in an average NE after NE correction of 0.016 / 0.007/ 0.007 mm in X / Y / Z directions, which were only 2.6% / 1.6% / 1.8% of the voxel size.

In previous studies, McDaid et al.^32^ and Mohamed et al.^33^ have reported ICC values in the range of 0.7 to 0.9 for identification of system- or patient-based distortions using manually selected landmarks^32,33^. The proposed method with automated quantification of MD outperformed the previous studies with superior reliability. In addition, Koff et al.^24^ evaluated the repeatability by measuring the geometric distortion without any sample material and with polyethylene material, resulting in displacements of 0.36 and 0.33 mm (87.8 and 80.5% of the in-plane resolution of 0.41 mm)^24^. In contrast, the proposed method exhibited extremely low residual error of 2.6, 1.6, and 1.8% of the voxel size.

### Influence of SNR on distortion evaluation

**Fig. 5:**
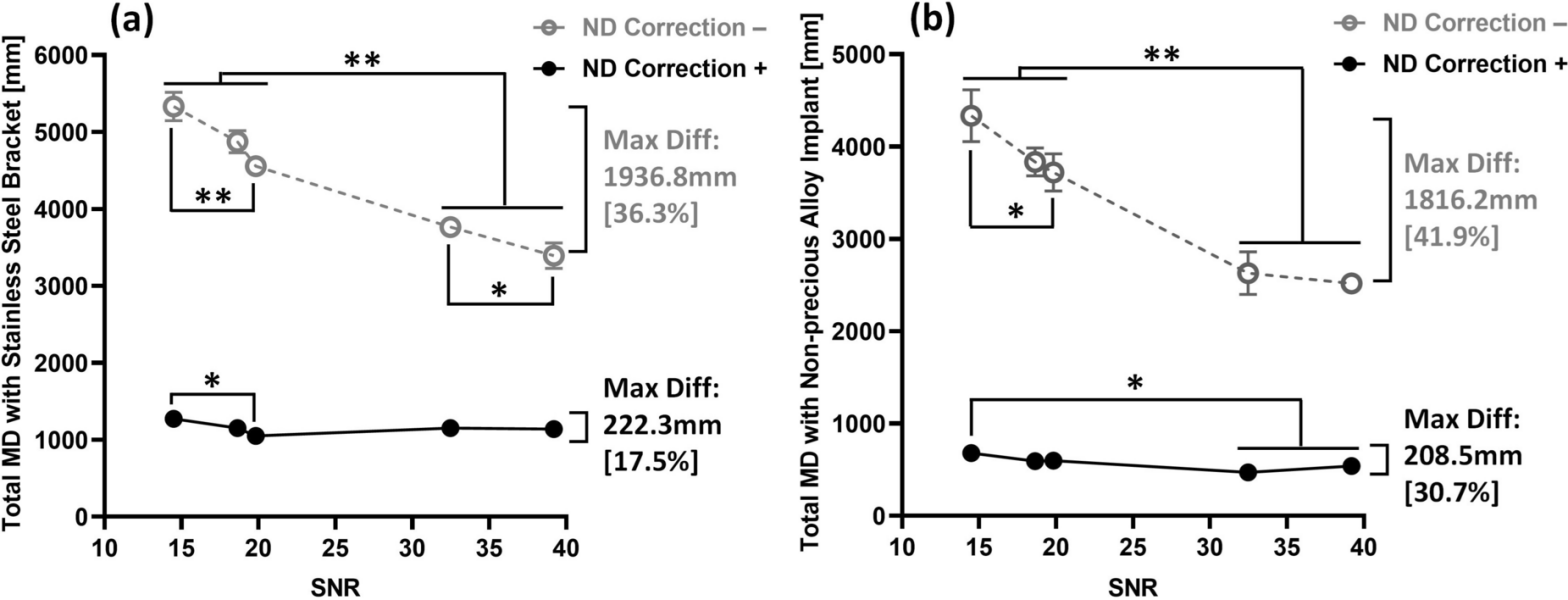
Comparison of total metal-induced distortions (MD) with different SNRs and different metallic devices without noise-induced error (NE) correction (grey) and with NE correction (black): (**a**) total MD with stainless-steel bracket, (**b**) total MD with non-precious alloy crown-supported implant. (* $p\le 0.05$, ** $p\le 0.001$).

For both materials, a significant influence of the SNR on the measured MD was noted (p-value ≤ 0.001; Fig. 5 “NE correction −”). With the increase of the SNR, the total MD decreased. The maximal differences in the detected MD were observed between VIBE1 and VIBE5 with 36.3% (1936.8 mm) / 41.9% (1816.2 mm) for stainless-steel bracket / non-precious alloy crown-supported implant, which were statistically significant (p-value ≤ 0.001). In contrast, NE correction resulted in a substantial reduction of the SNR influence by 89% for both materials (Fig. 5 “NE correction +”). The maximal differences in MD were reduced to 17.5% (222.3 mm) / 30.7% (208.5 mm) for both materials. The maximal crossing point-wise differences between protocols with NE correction also showed significant difference to those without NE correction (p-value ≤ 0.001). The detailed numerical results are provided in Supplementary Table S1.

### Estimation of the MD in artifact-affected regions

**Fig. 6:**
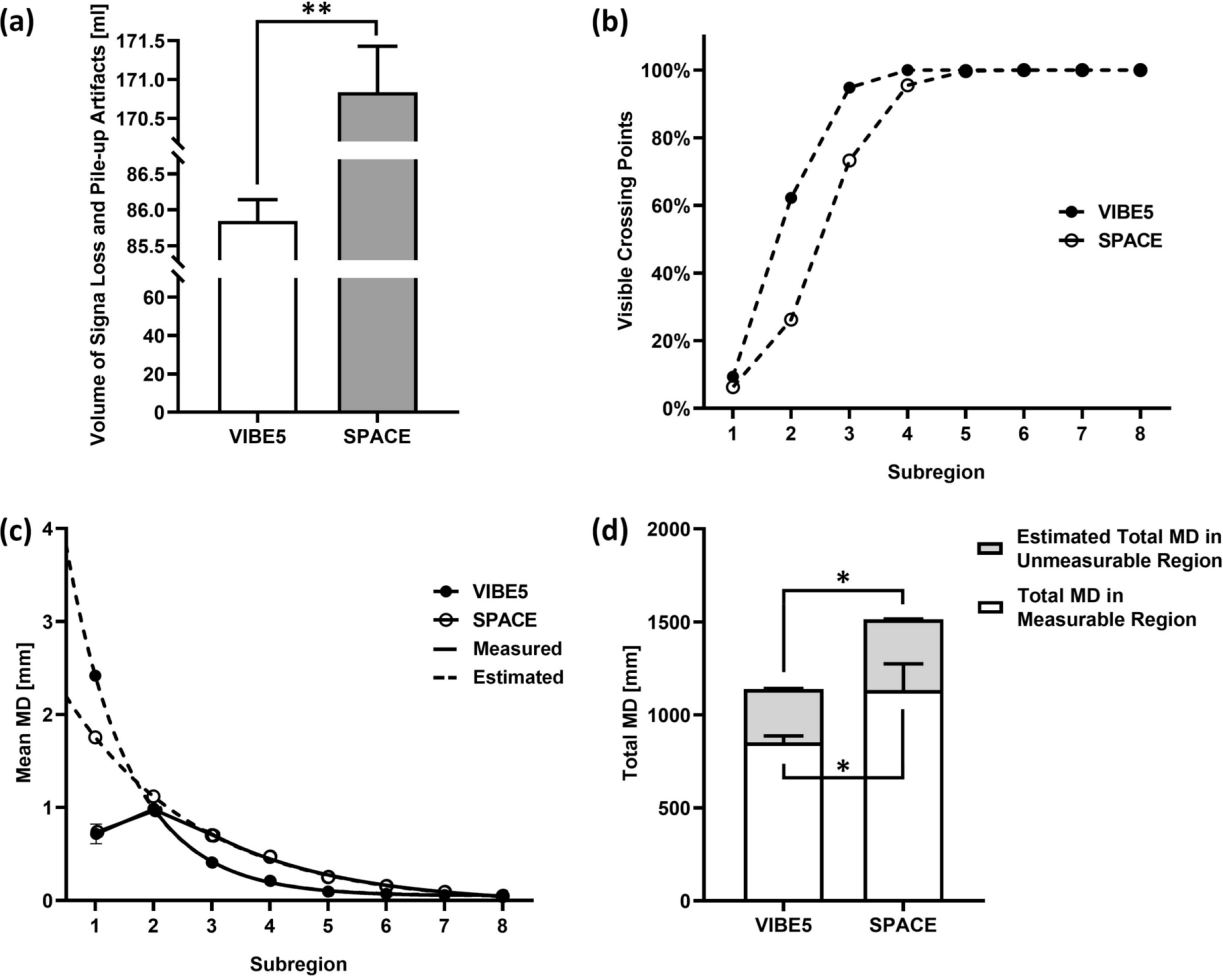
Comparison between VIBE5 and SPACE with stainless-steel bracket: (**a**) the volume of signal loss and pile-up artifacts; (**b**) the percentage of detected crossing points in subregions differed between both sequences due to the different volumes of artifacts; (**c**) measured (solid lines) and estimated (dash lines) mean MD per subregion; (**d**) total metal-induced distortions (MD) in measurable subregions (white) and estimated total MD in the artifact-affected subregions (gray). (* $p\le 0.05$, ** $p\le 0.001$).

To evaluate the importance of MD in artifact-affected regions, a prerequisite for a fair sequence comparison, two sequences with comparable SNRs but different artifact volumes (VIBE5 and SPACE; Fig. 6a) were compared. The artifact-affected volume of SPACE (170.8 ± 0.5 ml) was 98.5% larger than VIBE5 (85.8 ± 0.2 ml) when evaluating the stainless-steel brackets. As expected, the percentages of measurable crossing points in each subregion differed substantially between VIBE5 and SPACE (Fig. 6b). In subregion-1 9.4 / 6.3% of crossing points were evaluable in VIBE5 / SPACE and in subregion-2 62.2/ 26.2%. For VIBE5, the calculated / estimated mean MD in subregion-1 were 0.72 / 2.42 mm (Fig. 6c). For SPACE, the results for subregion-1 were 0.74 / 1.76 and for subregion-2 0.97 / 1.12 mm. The exponential growth fitting for both sequences obtained R^2^ > 0.99. Measured MD in regions with severely limited visibility of crossing points were 34.9 / 34.0% (274.13 ± 0.23 / 415.85 mm ± 0.54) lower than estimated MD by nonlinear fitting for VIBE5 / SPACE (Fig. 6d). The detailed numerical results are provided in Supplementary Table S2.

## Discussion

This study provides a precise and reliable method for a spatially and directionally coded distortions quantification in the presence of metal-induced-artifacts. Results showed high reliability with excellent intraclass correlation coefficient (≥ 0.99) and low mean errors in all directions (2.6%/1.6%/1.8% of voxel size in X/Y/Z direction). As a key finding, it identifies volume of signal loss/pile-up artifacts as well as SNR as significant confounder of measurement accuracy (p-value ≤ 0.001) and propose strategies to substantially decrease their influence to improve overall measurement accuracy (p-value ≤ 0.001). The provided method enables both: A comparison of distortions caused by different materials for a given sequence and comparison of sequences regarding their vulnerability to distortions for a given material. Finally, by adapting the phantom size, the method can be adopted to any part of the body and implant type.

Precise quantification of the effects of materials and sequences on the visualization of surrounding soft tissues is of importance as distortion may over- or underestimate the amount of pathological findings^34,35^ and affect therapy outcome due to registration errors^25,36^. The clinical relevance of this topic is significant, because an accurate and reliable method for quantification of metal-induced distortion may help to improve geometric accuracy of MRI to increase spatial accuracy in image-guided therapy (e.g., radiation oncology and neurosurgery)^37–39^ and real-time MRI-guided adaptive radiation therapy^40,41^. For instance, at a distance of 1–3 cm the brachytherapy dose gradient for a Iridium-192 source is 5.12% per mm resulting in a relevant dose effect on the tumor and organs at risk if small geometric errors are present^38^. The proposed method enables the identification of optimal sequence types / parameters for the lowest possible distortion. The use of optimized MRI protocols for minimal MD can improve treatment outcome of MRI-guided strategies (surgery, adaptive radiotherapy and brachytherapy) as spatial location of treatment site is specified more accurately compared to non-optimized protocols. The method allows in addition to further minimize MD and, thereby further improve accuracy. For given sequence parameters and implant it allows to precisely locate the metal within the signal loss artifact, which is of interest in MRI-guided brachytherapy as it improves the localization of the applicator. Finally, for the complete imaging volume a sequence and implant specific distortion profile can be created. These profiles can be applied to patient images in order to restore the correct spatial location of shifted anatomy due to the presence of metal. These advancements have the potential to improve treatment planning accuracy by ensuring precise tumor targeting and organ delineation, reduce safety margins to protect healthy tissues, and facilitate the reliable integration of MRI into surgery and radiooncology workflows, optimizing patient outcomes and minimizing treatment risks. Regarding the other factors that may induce geometric distortion, Glide-Hurst et al.^42^ state the range of system-induced frequency offset is around 5 ppm. The susceptibility of human tissues is on the order of − 10^–6^ ^43^, resulting in the chemical shift caused in the similar range. The tested protocols in this study adopted a high readout bandwidth (520 Hz/voxel) to compensate for the chemical shift induced by human tissues. Besides, the scanner-integrated 2D geometric correction were applied to correct the system-induced distortion. In contrast, the susceptibility of most medical metallic materials (including the materials used in this study) is in the range of 10^–4^ to 10^–3^ ^43^, leading to up to 150 ppm of frequency offset in the vicinity of metallic devices^44^. As a result, metal-induced geometric distortion, as an additional GD beside the system- and patient-induced GD, dominates in perturbating the geometric accuracy. Although the susceptibility artifacts can be reduced by using low field systems because the disturbance induced by metallic devices is proportional to the strength of the magnetic field, the SNR will also decrease proportionally. Since the demand on higher spatial resolution is increasing, using low field systems is not the optimal solution. Port and Pomper^45^ have analyzed the impact of the orientation of metallic devices in the magnetic field, and the results exhibit more severe susceptibility artifacts when the angle between the metallic device and the main magnetic field increases for gradient echo sequences, while spin echo sequence shows less sensitivity to the orientation of the metallic devices. In this study, the metallic devices were placed in the same direction as the in-vivo cases, though the proposed method was capable of quantifying MD regardless of the orientation of the metallic devices. Considering the huge difference between the severity of geometric distortion caused by metallic devices and system/patient, the MD profile obtained by the proposed method on phantom can be treated as an approximation of the in-vivo cases for physicians and medical physicists to be cautious about potentially regions and severity of distorted anatomical structures in the presence of metallic devices and approximately correct the geometric distortion caused by given metallic devices and sequences.

This study has several strengths. Sample materials used are frequently encountered in head and neck imaging, and phantom design can be adopted for other materials and other body regions. Next, 3D lattices design of the phantom allowed for automatic identification of crossing points and analysis incorporated all direction in space. In addition, accuracy of the method was verified using computed tomography scans.

Several previous studies proposed quantitative methods to evaluate the extent of signal loss and pile-up artifacts^2–6^ or evaluated the GD from B_0_ inhomogeneity without the presence of metal^30,46–48^. Some in-vivo studies evaluated patient-induced distortion in MR-guided radiotherapy or neurosurgery^11,12,39,49–51^. These studies calculate the distortion based on the distances between either the surface meshes generated from certain segmented organs^39,49,50^ or selected landmarks^12,39,51^ from target and reference images. These methods can only measure the distortion on the surface of target organs or at the positions of a limited number of landmarks. Only one study has examined volumetric GD produced by the metallic orthopedic materials in the whole FOV^24^. However, this study focused on the protocols optimized for total hip arthroplasty, where large slice thicknesses were employed instead of isotropic spatial resolution, also resulting in a limited number of analyzed slices. Furthermore, the impact of image acquisition parameters and artifact volume was not studied either. As a result, the method proposed by Koff et al.^24^ is of limited value for material or sequence comparison. In contrast, our study used a 3D lattice phantom with more than 9000 crossing points as the landmarks to analyze the protocols with isotropic resolution, which is typically used for navigated surgery, and the GD in three orthogonal directions was obtained simultaneously.

Due to the huge number of crossing points in the 3D lattice phantom, measuring the actual spatial locations of the crossing points on the phantom was not practical. Besides, considering the high accuracy of the proposed method with residual error down to 0.02 mm, the manufacturing error can be another influence to the accuracy of the quantification. This study employed the image-based quantification, which excluded the influence of other factors such as system- and patient-induced distortion, by directly comparing the images with and without a metallic device. Consequently, the metal-induced distortion was solely quantified. The proposed method was validated using CT images as baseline and achieved outstanding accuracy.

We also analyzed the impact of imaging parameters on the accuracy of the distortion quantification and corrected the error induced by the noise in the image. The difference in distortion among protocols with different SNR was comparable for both materials, which suggests that the influence of SNR is material independent. The proposed NE correction reduced the error induced due to SNR significantly (p-value ≤ 0.001). However, the proposed correction does not eliminate the influence of SNR completely. Nevertheless, the residual magnitude was comparably low with 0.024 mm / 0.022 mm mean differences for each crossing point for the stainless-steel bracket / non-precious alloy crown-supported implant. This corresponds to only 2.4% / 2.2% of the image resolution, highlighting the accuracy of the method. Therefore, protocols with a wide range of SNRs can be fairly compared with the proposed NE correction.

Finally, we provide a method for estimation of distortions in areas of signal-loss and pile-up artifacts. Depending on the studied material, this correction may not be necessary. With the non-precious alloy crown-supported implant for example, the percentages of measurable crossing points in all subregions for all protocols are above 95%. Thus, an estimation is not needed. However, with the stainless-steel bracket, it is impossible to accurately calculate the mean distortion in the subregions closest to the material due to signal loss and pile-up artifacts. Without calculating the estimated total MD in the artifact-affected region, however, it is not possible to compare the sensitivity of different sequences / sequence parameters in a fair manner as the number of evaluated crossing points vary substantially. This results in different measurement accuracies of the method for different sequences / sequence parameters. Therefore, the mean distortions in these subregions are projected using the exponential fitting of the measured mean distortion of other subregions. This allows a fair comparison of sequences with varying sensitivity to metal-induced artifacts.

This study still has several limitations. The proposed method is not able to remove the NE entirely, particularly for protocols with extremely low SNR, as significant differences were still observed between the total MDs of some protocols after NE correction. Besides, at least 3 repetitions of the measurements are required for each protocol to calculate the NE, and the accuracy of NE correction may be improved if more repetitions are performed, but more repetitions consume much longer scan time. In addition, using the MD profile acquired by the proposed method to correct MD in in-vivo images will be investigated in our future research.

## Conclusion

In conclusion, a precise and reliable method for a spatially and directionally coded distortions quantification in the presence of metal-induced-artifacts was developed with excellent intraclass correlation coefficient (≥ 0.99) and low mean errors in all directions (2.6%/1.6%/1.8% of voxel size in X/Y/Z direction). Furthermore, volume of signal loss and pile-up artifacts as well as SNR were identified as independent significant confounders (p-value ≤ 0.001) in metal-induced distortion quantification. Moreover, we propose correction methods for both confounders. MRI-guided treatment planning could benefit from this method in the future as it allows distortion evaluations of given materials as well as identification of optimal MRI sequences / sequence parameters for highest spatial accuracy even in the presence of metal implants independent of body region and implant type.

## Supplementary Information

Supplementary Information.

## Data Availability

All data generated and analyzed during this study are available from the corresponding author upon reasonable request.
