# Supplementary material for: 3D quantification of metal-induced geometric distortions in MRI
Source: Sci Rep. 2025 Feb 28;15:7195. doi: 10.1038/s41598-025-90645-5 (PMC11871036; doi:10.1038/s41598-025-90645-5)
Supplement: Supplementary file 1 — Supplementary Information. [file 41598_2025_90645_MOESM1_ESM.pdf]

3D quantification of metal-induced geometric distortions in MRI

Hao Li MSc[1], Ali C Özen PhD[2], Alexander Juerchott MD[1], Michael Breckwoldt MD PhD[1], Jessica Jesser MD[1], Dominik F Vollherbst MD[1], Daniel Schwarz MD[1], Martin Bendszus MD[1], Sabine Heiland PhD[1], Tim Hilgenfeld MD[1]*

[1]Department of Neuroradiology, University Hospital Heidelberg, Germany

[2]Department of Radiology, Medical Physics, Medical Center, Faculty of Medicine, University of Freiburg, Germany

Supplementary Table S1. The total metal-induced geometrid distortion (MD) and p-values between sequences, materials, and whether noise-induced error (NE) correction was performed. The highlighted p-values (underlined) represent significant differences.

				VIBE1	VIBE2	VIBE3	VIBE4	VIBE5
With stainless steel bracket	Without NE Correction	Mean MD (mm)		5330.6	4874.3	4556.7	3763.8	3393.8
		Max MD (mm)		5493.4	5014.5	4611.0	3867.7	3491.3
		Min MD (mm)		5130.3	4726.8	4500.5	3697.1	3201.5
		p-value	VIBE1	–	0.0148	0.0003	<0.0001	<0.0001
			VIBE2	–	–	0.1003	<0.0001	<0.0001
			VIBE3	–	–	–	0.0003	<0.0001
			VIBE4	–	–	–	–	0.0487
	With NE Correction	Mean MD (mm)		1273.9	1151.0	1051.6	1152.3	1139.1
		Max MD (mm)		1327.4	1181.5	1113.5	1229.0	1168.5
		Min MD (mm)		1221.7	1117.3	1000.4	1099.3	1097.3
		p-value	VIBE1	–	0.0864	0.0024	0.0907	0.0558
			VIBE2	–	–	0.1985	>0.9999	0.9983
			VIBE3	–	–	–	0.1897	0.2934
			VIBE4	–	–	–	–	0.9975
	p-value between with and without NE Correction			<0.0001	<0. 0001	<0. 0001	<0. 0001	<0. 0001
With non-precious alloy crown-supported implant	Without NE Correction	Mean MD (mm)		4333.5	3833.7	3718.3	2627.2	2517.3
		Max MD (mm)		4659.1	4002.1	3922.3	2801.1	2575.9
		Min MD (mm)		4153.6	3709.2	3518.1	2364.6	2414.7
		p-value	VIBE1	–	0.0756	0.0257	<0.0001	<0.0001
			VIBE2	–	–	0.9524	0.0002	<0.0001
			VIBE3	–	–	–	0.0005	0.0002
			VIBE4	–	–	–	–	0.9598
	With NE Correction	Mean MD (mm)		679.5	593.0	598.0	470.9	537.8
		Max MD (mm)		684.0	598.5	628.2	512.9	644.5
		Min MD (mm)		670.6	585.0	571.7	449.5	477.7
		p-value	VIBE1	–	0.2295	0.2742	0.0019	0.0254
			VIBE2	–	–	>0.9999	0.0565	0.6115
			VIBE3	–	–	–	0.0462	0.5385
			VIBE4	–	–	–	–	0.4439
	p-value between with and without NE Correction			<0. 0001	<0. 0001	<0. 0001	<0. 0001	<0. 0001

Supplementary Table S2. The total metal-induced geometrid distortion (MD) with and without compensation for signal loss and pile-up artifact region (SLPUA), and p-values of VIBE5 and SPACE with the stainless steel bracket. Noise-induced error correction was performed. The highlighted p-values (underlined) represent significant differences.

		VIBE5	SPACE
volume of SLPUA	Mean MD (ml)	85.8	170.8
	Max MD (ml)	86.0	171.5
	Min MD (ml)	85.5	170.5
	p-value between VIBE5 and SPACE	<0. 0001	
Without SLPUA compensation	Mean MD (mm)	851.3	1133.2
	Max MD (mm)	879.9	1295.0
	Min MD (mm)	812.1	1042.5
	p-value between VIBE5 and SPACE	0. 0280	
With SLPUA compensation	Mean MD (mm)	1139.1	1513.4
	Max MD (mm)	1168.5	1678.0
	Min MD (mm)	1097.3	1420.0
	p-value between VIBE5 and SPACE	0. 0118	
p-value between with and without SLPUA compensation		0.0006	0. 0304
